# 3D Bioprinted Vascularized Tumour for Drug Testing

**DOI:** 10.3390/ijms21082993

**Published:** 2020-04-23

**Authors:** Seokgyu Han, Sein Kim, Zhenzhong Chen, Hwa Kyoung Shin, Seo-Yeon Lee, Hyo Eun Moon, Sun Ha Paek, Sungsu Park

**Affiliations:** 1 School of Mechanical Engineering, Sungkyunkwan University, Suwon 16419, Korea; hsk0426@skku.edu (S.H.); zhong@skku.edu (Z.C.); 2 Department of Biomedical Engineering, Sungkyunkwan University, Suwon 16419, Korea; tpdlstjdtn@skku.edu; 3 Department of Korean Medical Science, School of Korean Medicine, Pusan National University, Yangsan 50612, Korea; julie@pusan.ac.kr; 4 Korean Medical Science Research Center for Healthy-Aging, Pusan National University, Yangsan 50612, Korea; 5 Graduate Training Program of Korean Medicine for Healthy-Aging, Pusan National University, Yangsan 50612, Korea; 6 Department of Pharmacology, School of Medicine, Wonkwang University, Iksan 54538, Korea; brainsw@gmail.com; 7 Department of Neurosurgery, Seoul National University Hospital, Seoul 03080, Korea; hyoes@hanmail.net (H.E.M.); paeksh@snu.ac.kr (S.H.P.); 8 Cancer Research Institute, Hypoxia Ischemia Disease Institute, Seoul National University, Seoul 03080, Korea; 9 Institute of Quantum Biophysics (iQB), Sungkyunkwan University, Suwon 16419, Korea; 10 Biomedical Institute for Convergence at SKKU (BICS), Sungkyunkwan University, Suwon 16419, Korea

**Keywords:** tumour microenvironment, bioprinting, blood vessel, angiogenesis, fibroblast

## Abstract

An in vitro screening system for anti-cancer drugs cannot exactly reflect the efficacy of drugs in vivo, without mimicking the tumour microenvironment (TME), which comprises cancer cells interacting with blood vessels and fibroblasts. Additionally, the tumour size should be controlled to obtain reliable and quantitative drug responses. Herein, we report a bioprinting method for recapitulating the TME with a controllable spheroid size. The TME was constructed by printing a blood vessel layer consisting of fibroblasts and endothelial cells in gelatine, alginate, and fibrinogen, followed by seeding multicellular tumour spheroids (MCTSs) of glioblastoma cells (U87 MG) onto the blood vessel layer. Under MCTSs, sprouts of blood vessels were generated and surrounding MCTSs thereby increasing the spheroid size. The combined treatment involving the anti-cancer drug temozolomide (TMZ) and the angiogenic inhibitor sunitinib was more effective than TMZ alone for MCTSs surrounded by blood vessels, which indicates the feasibility of the TME for in vitro testing of drug efficacy. These results suggest that the bioprinted vascularized tumour is highly useful for understanding tumour biology, as well as for in vitro drug testing.

## 1. Introduction

In vivo tumours form a microenvironment called a tumour microenvironment (TME), where tumour cells interact with adjacent stromal cells such as endothelial cells and fibroblasts and an extracellular matrix (ECM) [1]. This interaction is closely related to cancer progression, metastasis, and drug resistance [2]. For example, tumour cells and fibroblasts secrete angiogenic factors, such as the vascular endothelial growth factor (VEGF), leading to angiogenesis, which causes peripheral endothelial cells to form new blood vessels [3,4]. In turn, new blood vessels supply tumour cells with oxygen and nutrients, which are essential for tumour progression and metastasis [5]. In addition to VEGF, fibroblasts release various inflammatory cytokines such as interleukin 6 (IL-6) and transforming growth factor β1 (TGF-β1), to induce epithelial-mesenchymal transition (EMT) by increasing the expression of N-cadherin and vimentin in tumours [6,7]. Overexpression of these proteins is associated with a poor clinical outcome in cancer patients [8]. Thus, there is an urgent need to reconstitute the TME in vitro to better understand the biological properties of tumours and accurately predict their drug responses in vivo.

Several types of cancer cells can form spheroids called multicellular tumour spheroids (MCTSs) through self-aggregation [9]. MCTSs are commonly used to mimic the TME because of their similarity to tumours in vivo. For example, MCTSs larger than 200 μm are used to mimic cancer stem cell (CSC) niche due to a hypoxic environment in their core, where the stemness of CSCs is maintained [9,10,11,12]. To accurately observe quantitative drug responses, the size of the MCTSs should be controlled in a narrow range. Various methods have been developed to achieve this goal, including concave microwells [13,14]. However, most of these MCTSs lack endothelial and stromal cells, which play an important role in aggressive behaviours of tumour cells, such as angiogenesis and invasion [15]. Recently, microfluidic devices have been developed to recapitulate TME with perfusable vascular networks that exhibit angiogenesis [16,17,18,19]. However, because microfluidic devices are difficult to mass-produce, and their liquid handling for cell culture and drug testing in the devices is relatively complicated compared to those in microwell plates, their use in high-throughput screening (HTS) of anticancer drug efficacy is limited.

Bioprinting is a method for constructing complex three-dimensional (3D) biological structures via layer-by-layer printing of a bioink that comprises an ECM and cells, according to a computer-aided design [20]. Recently, several efforts have been made to reconstitute the TME of various cancers by 3D bioprinting technologies [21], including cervical cancer [22] and triple-negative breast cancer with fibroblasts [23]. Most recently, Langer et al. [24] bioprinted a heterotypic TME that consisted of patient-derived cancer cells, fibroblasts, and endothelial cells, closely mimicking the TME in vivo. The TME is highly useful for studying the effect of stromal cells on the response of TME to drugs. However, TMEs vary in size and may not be suitable for obtaining quantitative results regarding drug efficacy.

**Figure 1:**
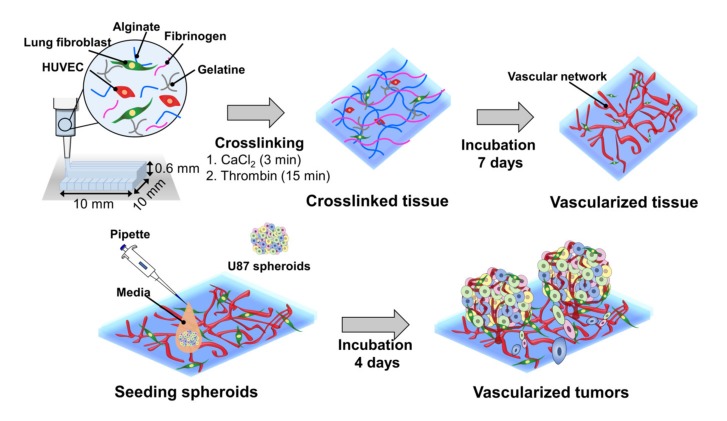
Combined use of bioprinted blood vessel layers and MCTSs to obtain uniform-sized vascularized tumours.

Herein, we report a bioprinting method for constructing a vascularized TME with a controllable size to accurately determine the responses of cancer to drugs (Figure 1). The blood vessel layer was constructed by culturing bioprinted human vascular endothelial cells (HUVECs) and lung fibroblasts (LFs) in gelatine/alginate/fibrinogen (GAF) hydrogel, until it formed blood vessels with lumens. Then, MCTSs were seeded into the blood vessel layer and incubated until the endothelial cells in the blood vessel layer migrated into the MCTSs and exhibited angiogenesis while some cancer cells invaded the blood vessel layer. The biological relevance of the biofabricated TME was verified by investigating the expression of blood vessel marker CD 31, as well as the expression of vimentin and N-cadherin. The feasibility of its application for testing of drug efficacy was demonstrated by observing the differential effects of anti-cancer drugs such as temozolomide (TMZ) and sunitinib (SU) on MCTSs with and without the blood vessel layer. Since vascularized tumour can form in microwell plates using our bioprinting method, it can be used for HTS of anti-cancer drugs efficacy.

## 2. Results and Discussions

### 2.1. 3D Bioprinting HUVEC/LF with GAF Hydrogel

**Figure 2:**
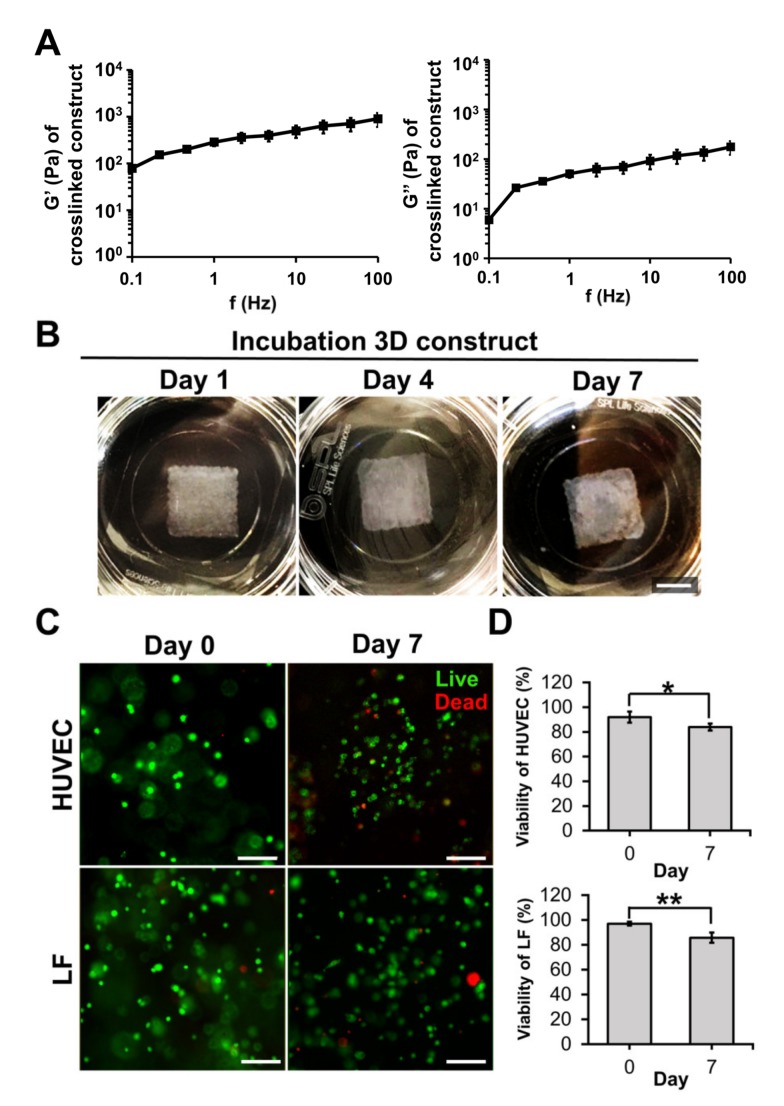
Mechanical properties and cell viability of the GAF hydrogel. (**A**) Storage (G’) and loss (G’’) moduli of the crosslinked GAF hydrogel measured from 0.1 to 100 Hz. (**B**) Images of cell-laden 3D constructs during incubation (one, four, and seven days). The scale bar represents 5 mm. (**C**) Live/dead staining image and (**D**) cell viabilities of HUVECs and LFs during incubation (zero and seven days). Live and dead cells were stained with calcein AM (green) and ethidium homodimer-1 (EthD-1) (red). The scale bar represents 100 μm. Student’s t-test; * *p* < 0.05, ** *p* < 0.01.

Bioink must have good mechanical properties for printing [25], as well as long-term cell culturing [26]. The storage and loss moduli of GAF hydrogels that were previously crosslinked with CaCl_2_ and thrombin were in the ranges of 0.7–9 and 0.06–1.7 kPa, respectively, as shown in Figure 2A. These values are similar to the storage and loss moduli of a previously reported gelatine-based hydrogel for long-term culturing of pancreatic islets cells [27]. When the bioprinted layer (10 × 10 × 0.6 mm^3^) of the GAF hydrogel encapsulating HUVEC/LF was incubated for seven days, its shape was maintained throughout the culturing, indicating that the hydrogel is physically stable for long-term culturing (Figure 2B).

The hydrogel can be printed in 24-well plates as well (Figure S1). GAF has high structural stability and is suitable for long-term cell culturing because GAF hydrogel is double crosslinked through the crosslinking of alginate and fibrinogen with CaCl_2_ and thrombin, respectively [28]. Gelatine was included in the GAF hydrogel because it has cell-binding motifs and can be physically crosslinked easily at low temperatures. Its crosslinked structure tended to dissolve quickly at 37 °C (data not shown). Thus, it was mixed with alginate and fibrinogen, which can be chemically crosslinked [29]. Alginate can rapidly chemically crosslink with divalent cations such as Ca^2+^, while fibrinogen can be polymerized to fibrin with thrombin.

Bioink must be biocompatible with cell adhesion and growth. Immediately after the printing, the viability of the HUVECs and LFs was greater than 90% (Figure 2C,D). It remained above 80% even on day seven. The viability is comparable or superior to that of HUVECs and fibroblasts cultured on other hydrogels, such as the poly(ethylene glycol)-polycaprolactone hydrogel or alginate dialdehyde-gelatine [30,31]. The results suggest that the GAF hydrogel is suitable for bioprinting as well as for growing HUVECs and LFs.

### 2.2. Microvessel Formation in GAF Hydrogel Layer Encapsulating HUVEC/LF

**Figure 3:**
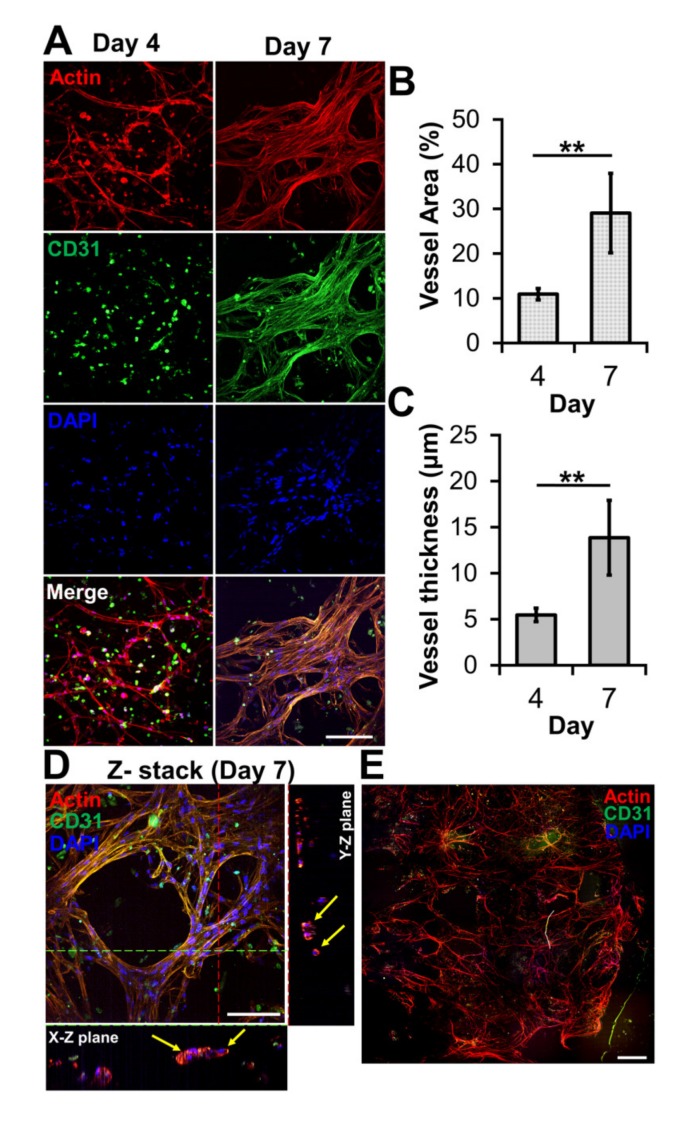
Microvessel formation in GAF hydrogel layer encapsulating HUVEC/LF. (**A**) Confocal images of microvessels in the layer stained with phalloidin (red, actin), Alexa Fluor 488-conjugated anti-CD31 antibody (green, blood vessel), and DAPI (blue, nucleus) on days four and seven. The scale bar represents 100 μm. (**B**) Area and (**C**) thickness of microvessels in the layer. Each analysis was performed by randomly selecting nine different regions in two printed layers. Student’s *t*-test: ** *p* < 0.01. (**D**) Cross-sectional images of microvessels in the bioprinted layer, showing lumens. The yellow arrow indicates lumen formation. The scale bar represents 100 μm. (**E**) Stitched images of microvessels in the entire layer on day seven. The scale bar represents 500 μm.

The ability of bioprinted hydrogel layers containing HUVECs and LFs to form blood vessels was determined by staining with DAPI for the nucleus, the anti-CD31 antibody for a blood vessel, and phalloidin for actin. On day four, the actin staining revealed capillary networks (Figure 3A) with a low expression of CD31, which is a protein expressed in endothelial intercellular junctions when endothelial cells form capillary tubes. The images showed that the capillary networks were not yet fully developed. However, on day seven, the actin staining revealed well-developed capillary networks with a high expression of CD31 (Figure 3A). This was confirmed by measuring the area and thickness of blood vessel in the layer, indicating that the blood vessels on day seven were thicker and larger than those on day four (Figure 3B,C). When the GAF hydrogel encapsulating only HUVECs was printed, microvessels were not observed until day seven, indicating that LFs are required for HUVECs to form vascularized tissues (Figure S2).

Lumens are indicators of a mature blood vessel. Orthogonal sectioning of the 3D image revealed that lumens formed in the microvessels on day seven (Figure 3D). Their sizes ranged from 10 to 25 μm, which is similar to the range of sizes of lumens in microvessels formed via a previously reported bioprinting method [32]. The stitched image indicated that microvessels were present in most of the printed layer (Figure 3E). Compared with other biofabrication methods for vascularized tissue that use either sacrificial lumen structures or preformed blood vessel channels [33,34], our method has the advantage of being able to produce large open vascularized tissue easily and rapidly.

### 2.3. Seeding Uniform-Sized MCTSs onto Vascularized Tissue

**Figure 4:**
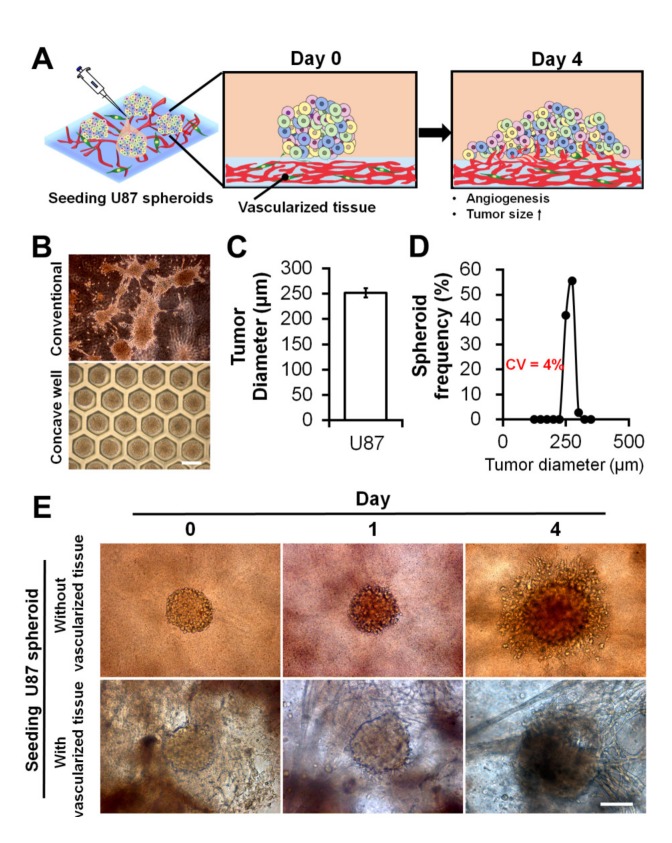
Uniform-sized U87 MCTSs and their seeding onto GAF hydrogel layers with and without preformed vascularized tissues. (**A**) Schematic describing the morphological changes and angiogenesis of MCTSs seeded onto the vascularized tissue. (**B**) Optical images of MCTSs formed on petri dishes and concave wells on day three. The scale bar represents 400 μm. (**C**) Diameter and (**D**) size distribution of MCTSs formed in concave wells. (**E**) Optical images of MCTSs seeded onto GAF hydrogel layers with and without preformed vascularized tissues on days zero, one, and four. The layer with vascularized tissues was constructed by incubating the bioprinted GAF hydrogel encapsulating HUVECs and LFs for seven days in the media, and the layer without vascularized tissues was constructed by printing the bare GAF hydrogel. The scale bar represents 200 μm.

MCTSs with a narrow size distribution are a prerequisite for obtaining a statistically robust drug response. Once the vascularized tissue was obtained, we seeded uniform-sized MCTSs onto the tissue to investigate the effects of the blood vessel on the progression, angiogenesis, and drug response of the MCTSs, as depicted in Figure 4A. In this regard, by culturing U87 cells in non-adherent concave wells for three days (Figure 4B), MCTSs with an average diameter of approximately 250 μm (Figure 4C) and low variances were obtained (coefficient of variation: approximately 4%) (Figure 4D). Without using the concave microwells, the size of the MCTSs could not be controlled (Figure 4B and Figure S3).

On day one, after seeding several MCTSs onto the GAF layers with and without vascularized tissues, there appeared to be no difference in the size of the MCTSs between the layers. However, on day four, the MCTSs on the layer with vascularized tissue appeared larger than those on the layer without vascularized tissue (Figure 4E), implying that the blood vessel may have accelerated the growth of the MCTSs. Due to the thick layer of bioprinted tissue, optical imaging did not ensure an exact measurement of the size of the MCTSs.

### 2.4. Effects of Vascularized Tissue on Growth, Angiogenesis, and EMT of MCTSs

**Figure 5:**
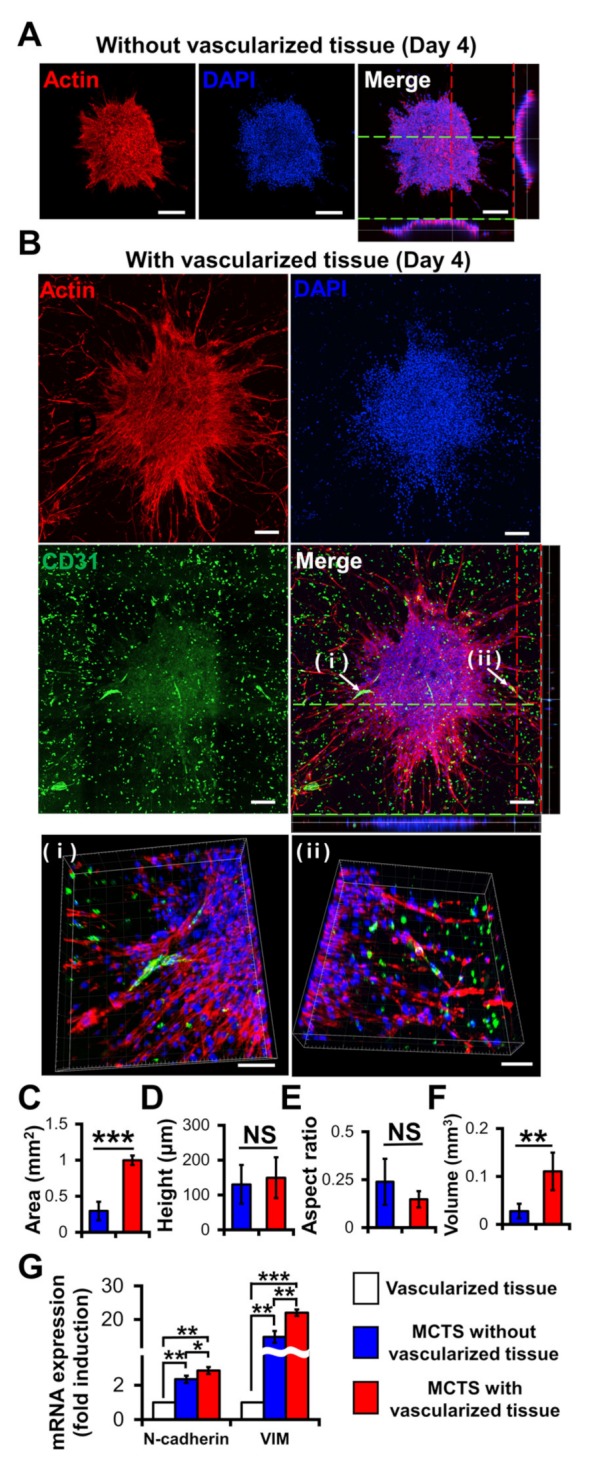
Effects of vascularized tissues on the growth and morphology of U87 MCTS. Confocal images of MCTSs without (**A**) and with (**B**) vascularized tissues. The scale bar represents 200 μm or (**B** (ⅰ,ⅱ)) 100 μm. (**C**) Area, (**D**) height, (**E**) aspect ratio and (**F**) volume of tumours were measured for MCTSs with/without vascularized tissues. Data were obtained using 4–6 MCTSs in two tissues for each sample. Student’s t-test; ** *p* < 0.01, *** *p* < 0.001; “NS” denotes “not significant”. (**G**) mRNA Expressions of N-cadherin and vimentin. All experiments were performed in triplicate. Student’s t-test; * *p* < 0.05, ** *p* < 0.01, *** *p* < 0.001.

Compared with the MCTSs on the 3D construct without vascularized tissues (Figure 5A), those on the 3D construct with vascularized tissues exhibited more evident actin protrusions (Figure 5B) indicating that the migratory activity of cancer cells increased [35]. The 3D images indicated that neovascularized structures infiltrated into MCTSs from the vascularized tissues (Figure 5B(i,ii)). Although there was no significant difference in height (Figure 5D) or aspect ratio (Figure 5E) between the two groups, the latter was about 3.8 times larger than that without the former (Figure 5F). These results indicate that the latter grew faster in the X and Y directions than the former. Taken together, blood vessels and fibroblasts in TME play important roles in the proliferation, angiogenesis, and migration of tumours [35,36].

Interestingly, the neovascularized structure seemed to be mostly disconnected as shown in Figure 5B. A similar observation was previously reported [36]. The aggressive growth of malignant cell population and their overexpression of pro-angiogenic factors can lead to the development of disorganized blood vessel networks. In disconnected vascular networks, oxygen supply is limited lead to induced micro-regional hypoxia, which is one of the key features in TME.

To determine the biological relevance of the neovascularized MCTSs, the expression of EMT markers such as N-cadherin and vimentin in MCTSs with and without vascularized structures was measured using RT-PCR [37]. Since the MCTSs with vascularized tissue contained U87, HUVECs and fibroblasts while those without vascularized tissue did not contain TME cells, we checked expression of N-cadherin and vimentin in vascularized tissue. The expression of both N-cadherin and vimentin in the vascularized tissue was significantly lower than that in the MCTSs without the vascularized tissue, suggesting that the MCTSs spontaneously increase the expressions of both protein. This is not surprising because 3D spheroids are known to have higher expression of both proteins than 2D cell monolayers. The expression of each protein in MCTSs with the vascularized tissue was approximately 1.5 times higher than that in MCTSs without the vascularized structures (Figure 5G). Since both genes are involved in angiogenesis and invasion of cancer cells in TME [38], the increase in the expression of both proteins in the MCTSs seeded onto the vascularized tissue might have contributed to the formation of neovascularized structures and spiked actin structures, an indicator for cell invasion. When the neovascularized structures formed into MCTSs, some growth factors released from the neovascularized structures might have increased the expression of both proteins. Taken together, this result supports that TME was well recapitulated in the bioprinted vascularized tumour.

### 2.5. Effects of TMZ and SU on Vascularized MCTSs

**Figure 6:**
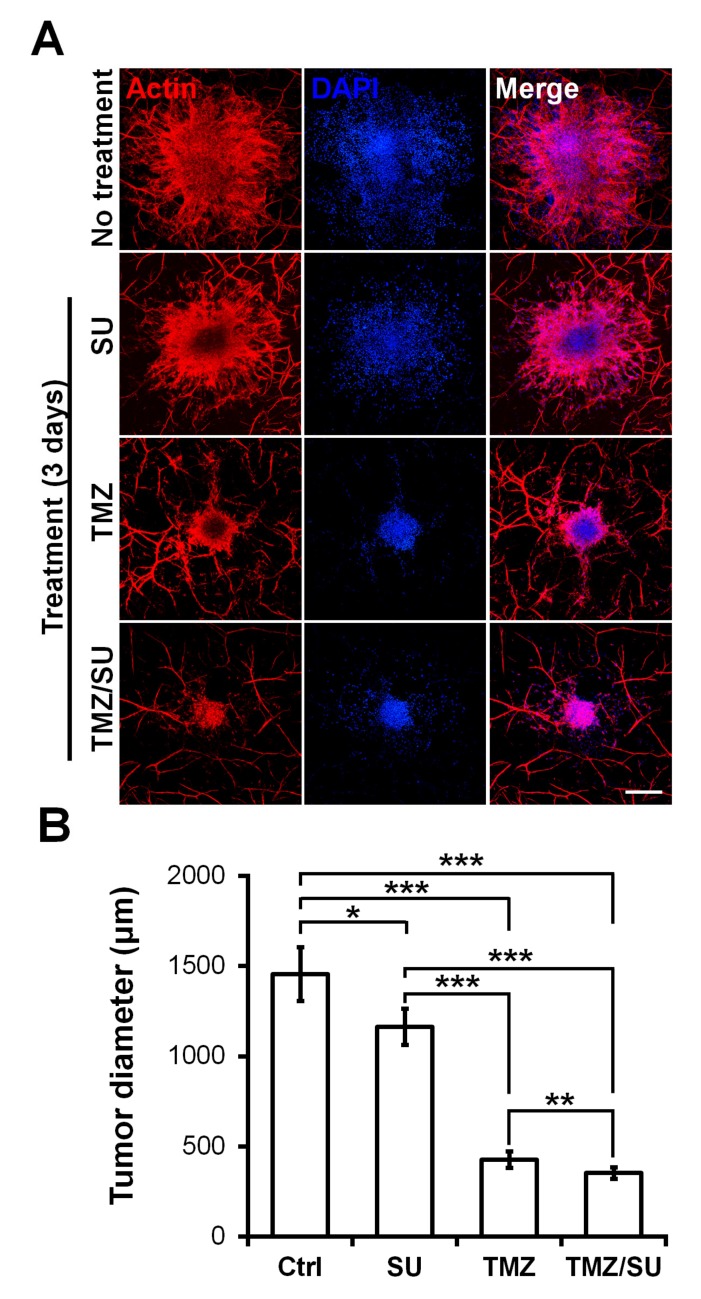
Synergistic effect of the anti-cancer drug TMZ and the blood vessel inhibitor SU on U87 MCTSs on vascularized tissues. (**A**) Actin (red), DAPI (blue), and merged image of MCTSs on vascularized tissue treated with TMZ (500 μM), SU (50 μM), or TMZ (500 μM)/SU (50 μM) for 72 h. The scale bar represents 500 μm. (**B**) Diameter of the drug treated MCTSs. The data were obtained for 4–6 MCTSs on two different bioprinted layers for each drug treatment. Student’s t-test; * *p* < 0.05, ** *p* < 0.01, *** *p* < 0.001.

To demonstrate the feasibility of the MCTSs biofabricated on the vascularized tissues for drug testing, the MCTSs were treated with the traditional anti-cancer drug TMZ, the blood vessel inhibitor SU, or with TMZ and SU together. On day zero, vascularized MCTSs were treated with TMZ and SU separately or together for three days. Actin/DAPI staining confirmed the size of the U87 MCTSs (Figure 6A). The size of MCTS was significantly reduced for the SU- and TMZ-treated samples compared with the untreated sample (Figure 6B). Additionally, there was a significant difference in size between the MCTSs treated with the different drugs. Compared with the treatments involving only TMZ or SU, those involving both TMZ and SU together further reduced the tumour size. These results are similar to the results of combination therapy involving TMZ and SU with U87 cells inserted into mice. A synergistic effect of TMZ and SU was observed when they were applied together, compared with the case where only TMZ was applied [39]. Therefore, the results observed in this microenvironmental tissue are analogous to the in vivo results. Furthermore, because MCTSs with a uniform size were cultured at the beginning, the drug-screening results can be predicted based only on the size [40,41]. A size reduction of the MCTSs was observed in the isolinderalactone-treated samples as well (Figure S4). Isolinderalactone is an extract from *Lindera aggregata*, which is a traditional Chinese herbal medicine [42]. The result suggests that the bioprinted blood vessel layer is suitable for interaction with multiple MCTSs (approximately 4–7) and can be widely used for testing various anti-cancer drugs.

## 3. Materials and Methods

### 3.1. Cell Culture

HUVECs, a human glioblastoma cell line U87 MG (U87), and LFs of human origin were obtained from American Type Culture Collection (ATCC, Bethesda, MD, USA). HUVECs in passage five were cultured in endothelial growth medium-2 (EGM-2) (Lonza, Basel, Switzerland) at 37 °C in a CO_2_ incubator. LFs in either passage five or six were cultured in a fibroblast growth medium (FGM-2) from Lonza at 37 °C in a CO_2_ incubator. U87 cells were cultured in minimum essential medium from Life Technologies (Carlsbad, CA, USA) supplemented with 10% fetal bovine serum from HyClone Laboratories, Inc. (Logan, UT, USA) and 1% penicillin (Life Technologies) at 37 °C in a CO_2_ incubator.

### 3.2. Preparation of Cell-GAF Bioink

A gelatine solution was prepared by dissolving 20% (*w*/*v*, final conc.) in a 0.9% (*w*/*v*) NaCl_2_ solution, and an alginate solution was prepared by dissolving 4% (*w*/*v*) in a 0.9% (*w*/*v*) NaCl_2_ solution. The two solutions were mixed in a volume ratio of 2:1 at 60 °C for 1 h and then at room temperature (RT) for 2 h. A fibrinogen solution was prepared by dissolving 4% (*w*/*v*) fibrinogen (Sigma–Aldrich, St. Louis, MO, USA) in phosphate-buffered saline (PBS, pH 7.4), followed by heating at 37 °C for 1 h. The fibrinogen solution was used to suspend freshly harvested HUVECs and LFs at the same density of 4 × 10^6^ cells/mL. Then, the gelatine/alginate hydrogel solution and cell-suspended fibrinogen solutions were mixed in a ratio of 3:1, so that the final concentrations of gelatine, alginate, and fibrinogen were 10%, 1%, and 1%, respectively, while the cell density of HUVECs and LFs was 1 × 10^6^ cells/mL each.

### 3.3. Bioprinting of Blood Vessel Layer

The freshly prepared GAF hydrogel encapsulating HUVECs and LFs was drawn into a 10-mL syringe (HSW, Tuttlingen, Germany) having a tip diameter of 250 μm (Nordson EFD, East Providence, RI, USA). The syringe was cooled in a refrigerator at 4 °C for 2 min and then mounted onto a bioprinter (INVIVO) (ROKIT Healthcare, Seoul, Korea). The temperatures of the dispenser and bed in the printer were set as 23 and 19 °C, respectively. Petri dishes were treated with 1% [*v*/*v*] polyethyleneimine (PEI) for 30 min and 0.1% [*v*/*v*] glutaraldehyde for 30 min to allow adhesion between the hydrogel and surfaces [43]. A HUVEC/LF construct with a cuboidal shape (10 × 10 × 0.6 mm^3^) was printed in a layer-by-layer manner into the petri dish. After the bioprinting was complete, the construct in the petri dish was filled with 1 mL of 3% (*w*/*v*) CaCl_2_ in deionized distilled water (DDW) and incubated at RT for 3 min to crosslink the alginate. The crosslinked construct was washed three times with PBS (pH 7.4). For crosslinking of fibrinogen, 2 U/mL (final conc.) of thrombin in PBS was added to the petri dish, and the dish was incubated at RT for 15 min (Figure 1). The construct was washed three times with PBS again and cultured in EGM-2 at 37 °C in a CO_2_ incubator for seven days to induce blood vessel formation.

### 3.4. Preparation of MCTSs and Their Seeding onto Bioprinted Blood Vessel Layer

A volume of 1 mL of freshly harvested U87 cells at a concentration of 3 × 10^6^ cells/mL were seeded into concave microwells (number of wells: 853) having a top diameter of 400 μm (StemFIT3D) (Microfit, Seongnam, Korea) and cultured with medium exchange every day at 37 °C in a CO_2_ incubator for three days. Finally, the individual spheroids in the wells were gently collected and pooled.

EGM-2 was carefully removed from the bioprinted construct in a petri dish that had been incubated for seven days. Next, 4–6 spheroids were seeded onto the top of the construct. Then, the construct with spheroids was incubated at 37 °C with 5% CO_2_ for 2 h until the spheroids were attached to the construct.

### 3.5. Rheological Measurement of GAF Hydrogel

The rheological properties of the crosslinked GAF hydrogel were measured using a rheometer (AR 2000ex, New Castle, IN, USA) with the mode of frequency sweep from 0.1 Hz to 100 Hz at 1% strain and 25 °C. The measurement was performed twice for each sample.

### 3.6. Immunostaining

Bioprinted constructs were carefully washed with PBS, fixed in 4% paraformaldehyde for 15 min at RT, and permeabilized with PBS containing 0.15% (*v*/*v*) Triton X-100 (Sigma–Aldrich) for 20 min at RT. They were then blocked with 3% bovine serum albumin for 1 h at RT. The samples were incubated overnight with Alexa Fluor 488-conjugated mouse CD31 antibody (Biolegend, San Diego, CA, USA) at 4 °C. Then, the F-actin of the cells in the constructs was stained with rhodamine phalloidin (Invitrogen), while the nuclei of the cells were stained with 4′,6-diamidino-2-phenylindole (DAPI) (Sigma–Aldrich).

### 3.7. Imaging and Analysis

A bright-field image was captured using an inverted microscope (Eclipse TE2000-U, Nikon). Fluorescent images were collected using spinning-disc confocal microscopy (Andor Dragonfly 302, Oxford Instruments, Concord, MA, USA). For quantification, Image J (NIH, Bethesda, MD, USA) was used. To measure the length and area of the microvessels and the area of the tumour, a binary image was produced, and z-stack images were projected into a single image with the maximum value. The height of the spheroids was calculated the by stacking 3D z-sectioned images of fluorescently labelled spheroids. The volume was measured by the calculation formula [44] which V = (W^2^ × L)/2, where V is tumour volume, W is tumour width and L is tumour length. The widths and heights were measured from the confocal images.

### 3.8. RT-PCR

The total RNA was extracted from the cells and purified using a RNeasy mini kit (Qiagen, Hilden, Germany). Then, complementary DNA (cDNA) fragments were synthesized from the extracted RNA using a 1st Strand cDNA Synthesis System (LeGene Biosciences, San Diego, CA, USA). Primers for genes encoding GAPDH, N-cadherin, and vimentin (Table 1) were designed by Bioneer Co. (Daejeon, Korea). *gapdh* was used to normalize the gene expression of the genes. iQ SYBR Green premix (Bio-Rad, Hercules, CA, USA) was used to measure the increasing amount of amplified DNA. The gene expression was checked via qRT-PCR using a Lightcycler® Nano System (Roche, Germany).

### 3.9. Drug Test

TMZ and SU were purchased from Sigma–Aldrich. TMZ and SU stock solutions in dimethyl sulfoxide (DMSO) (0.04%, final conc.) were diluted in EGM-2 before use. After the spheroid was attached to the construct, treatment was performed using TMZ (500 μM), SU (50 μM), or a combination of the two drugs (TMZ/SU; TMZ 500 μM, SU 50 μM). Then, the constructs were incubated for three days before imaging.

### 3.10. Statistical Analysis

All the data are represented as the mean ± standard deviation. Comparisons of the mean values between two groups were performed using Student’s t-tests. The levels of statistical significance were set as * *p*< 0.05, ** *p* < 0.01, and *** *p* < 0.001.

## 4. Conclusions

We developed a biofabrication method for a TME by combining an MCTS-forming technique with a bioprinting one. Through this combination, we obtained uniform-sized vascularized MCTSs. These MCTSs grew rapidly and exhibited angiogenesis and EMT. They are highly useful for obtaining accurate drug responses because of their narrow size distribution. For drug testing, their viability arises from the size of the spheroids, as spheroids with a uniform size were seeded on the structure. This platform can easily be used for drug-testing patient-derived cells (PDCs) by seeding spheroids of PDCs on preformed vascularized tissues.

## Figures and Tables

**Table 1:** Primers for genes encoding GAPDH, N-cadherin, and vimentin.

Gene Name	Primer Sequence (5′-3′)
*gapdh*	F: AGGTGGTGAAGCAGGCGTCGGAGGG
	R: CAAAGTGGTCGTTGAGGG
*cdh2*	F: CCCATACACCAGCCTGGAAC
	R: ACTAACCCGTCGTTGCTGTT
*vim*	F: CCGCACATTCGAGCAAAGAC
	R: ATTCAAGTCTCAGCGGGCTC

## Supplementary Materials

Supplementary materials can be found at https://www.mdpi.com/1422-0067/21/8/2993/s1.
