# Supplementary material for: 3D Bioprinted Vascularized Tumour for Drug Testing
Source: Int J Mol Sci. 2020 Apr 23;21(8):2993. doi: 10.3390/ijms21082993 (PMC7215771; doi:10.3390/ijms21082993)
Supplement: Supplementary file 1 [file ijms-21-02993-s001.pdf]

Supplementary Information

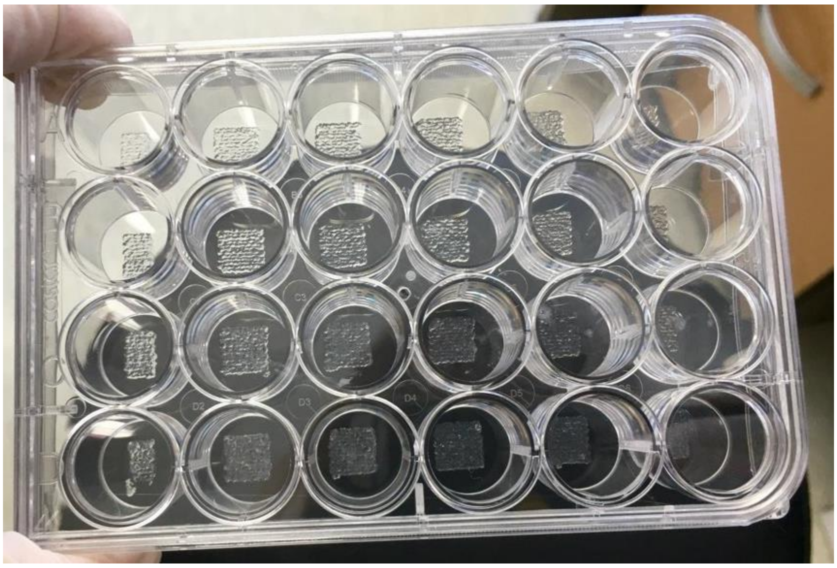

Figure S1. Bioprinted vascularized tissue onto conventional 24 well plate.

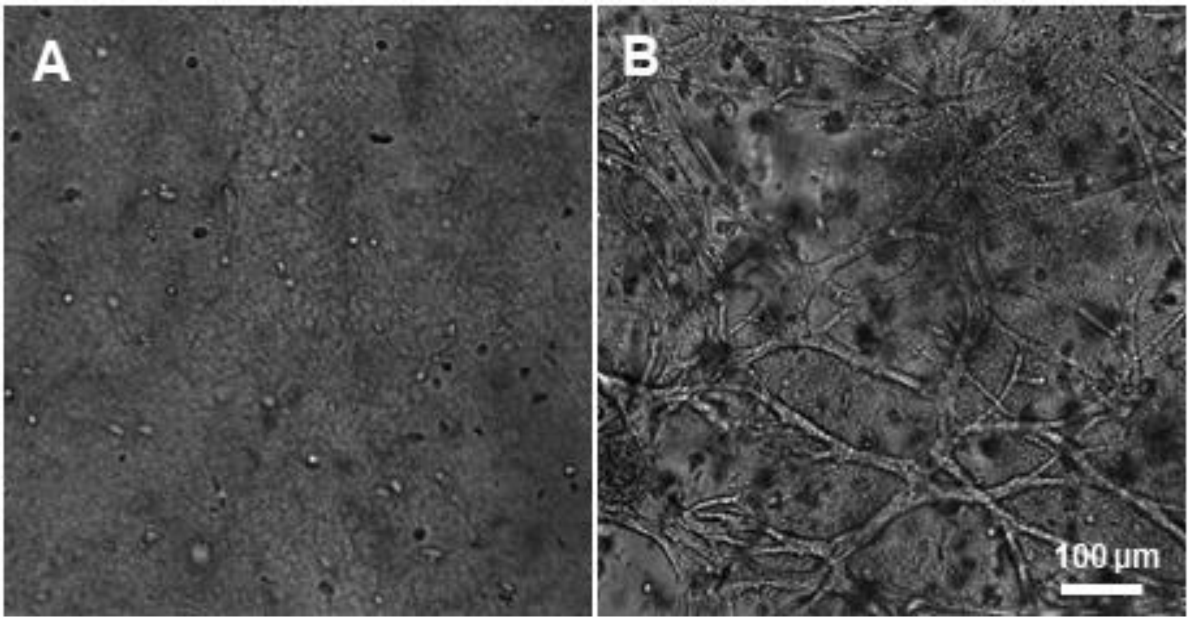

Figure S2. HUVEC forming vascular network only presence of lung fibroblasts (**A**) Optical image of HUVEC in bioprinted gel/alg/fib without lung fibroblast on day 7. (**B**) Optical image of HUVEC forming vascular network in bioprinted gel/alg/fib with lung fibroblast on day 7.

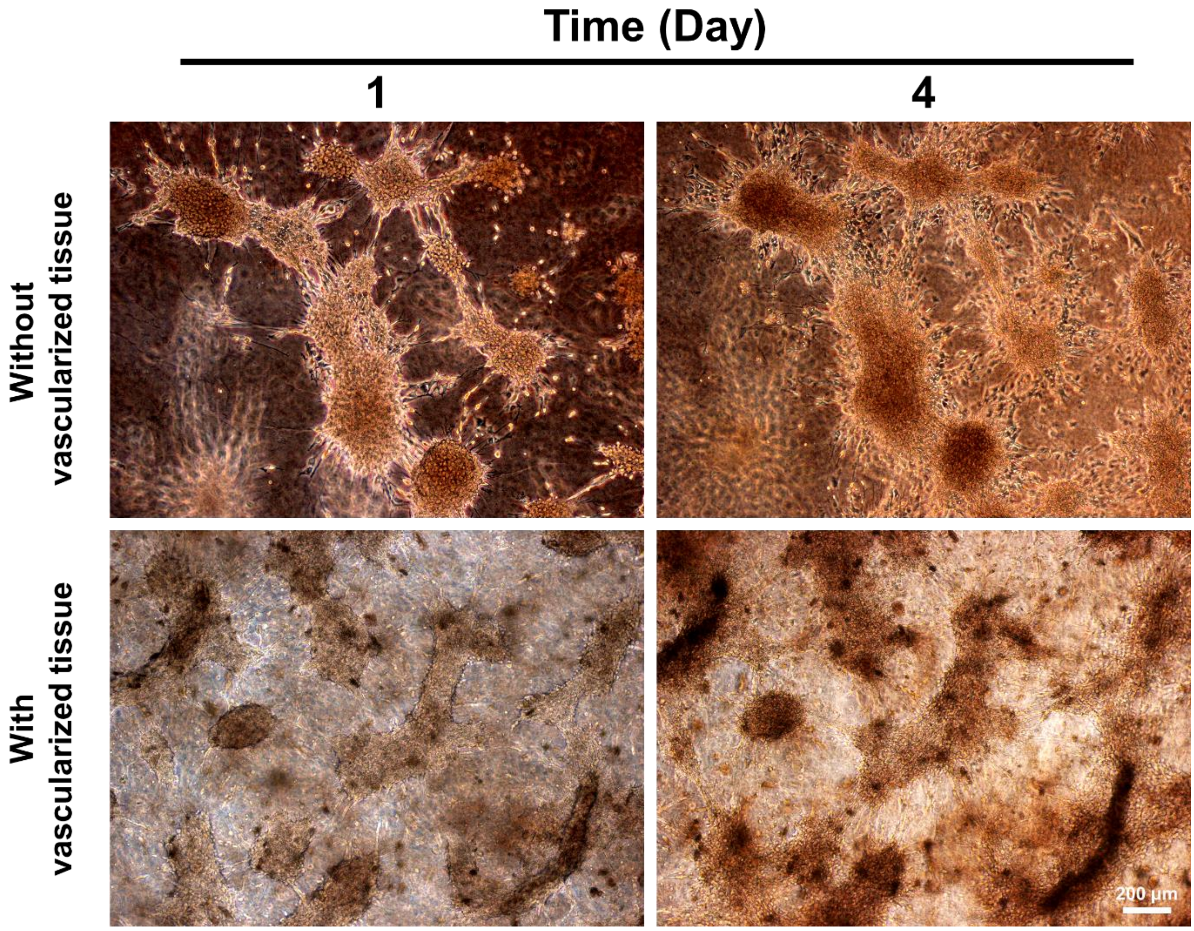

Figure S3. Optical image of U87 aggregated on bioprinted tissue with/without vascularization on day 1 and 4.

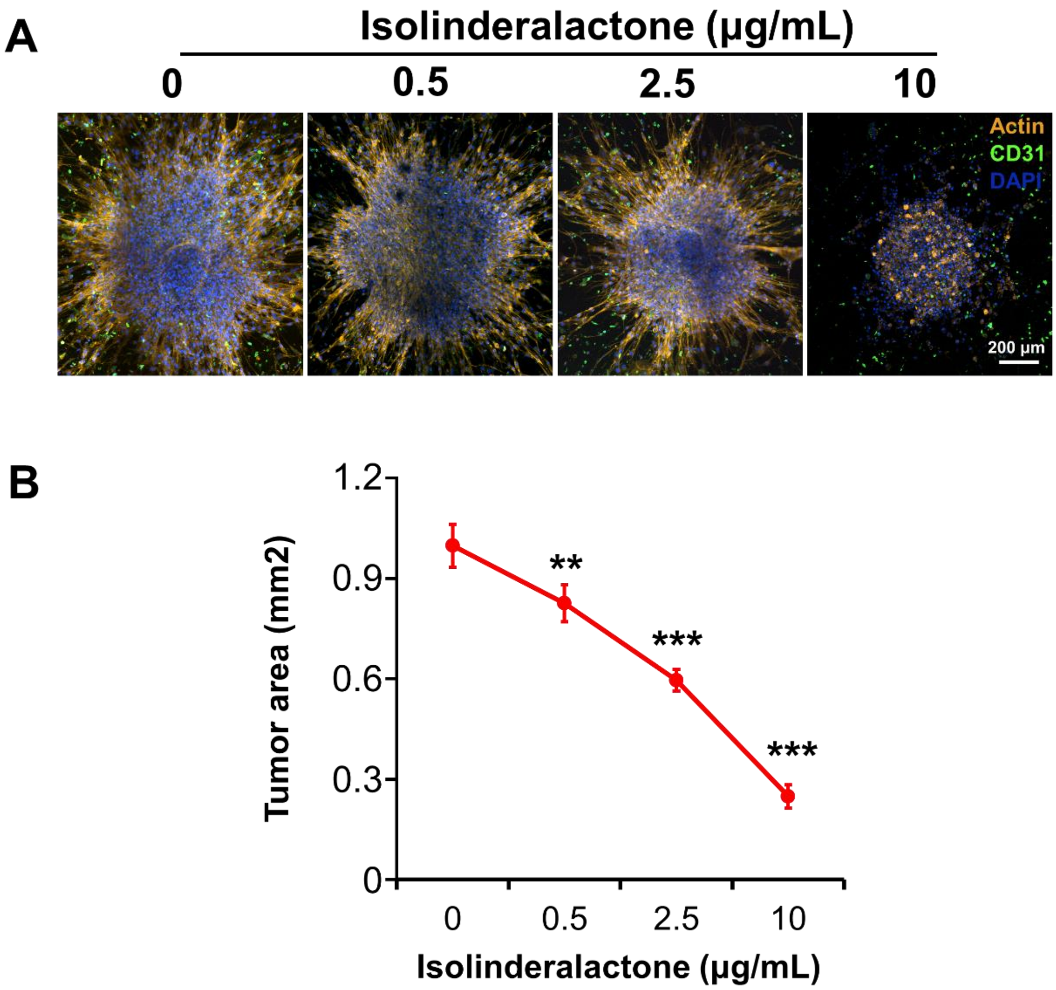

Figure S4. (**A**) Confocal image of U87 spheroid on vascularized tissue treated with different concentration (0, 0.5, 2.5,10 µg/mL) of isolinderalactone. (**B**) Tumor area of U87 spheroid treat with different concentration of isolinderalactone. N = 4-5; Student's t-test, $^{**}p < 0.01$, $^{***}p < 0.001$.
